# 1999. Assessment of Communication Strategies 1 to 2 Years After Completion of a Vaccine Hesitancy Communication Training Program During Residency

**DOI:** 10.1093/ofid/ofad500.126

**Published:** 2023-11-27

**Authors:** Shanna M Barton, Aaron W Calhoun, Yana Feygin, Gary S Marshall

**Affiliations:** Norton Children's and the University of Louisville School of Medicine, Louisville, KY; University of Louisville, Louisville, Kentucky; University of Louisville, Louisville, Kentucky; Norton Children's and University of Louisville School of Medicine, Louisville, KY

## Abstract

**Background:**

We previously showed (*J Pediatr* 2022;241:203; *Open Forum Infect Dis* 2022;9(2):ofac492.626) that residents trained in a structured communication strategy called AIMS (Announce, Inquire, Mirror, Secure) demonstrated specific behaviors of interest during live and virtual encounters with standardized patients (SPs) portraying vaccine-hesitant parents. The current study was conducted to determine if those behaviors were prioritized long after completion of training.

**Methods:**

In the original studies, blinded Pediatrics and Medicine-Pediatrics residents were randomized to AIMS training (AIMS Group) or standard of care training (Control Group; Figure 1); blinding was maintained after training and until the time of the current study. Subjects were invited to complete an online survey wherein 10 possible communication behaviors were ranked from highest priority to lowest priority (Figure 2). Respondents were considered to have prioritized AIMS behaviors if they ranked each of the 3 AIMS items in the top 5; the ability of this measure to detect prioritization of AIMS behaviors was assessed by surveying unblinded residents who underwent AIMS training in the prior 2 weeks (Validation Group). Differences between groups were assessed using Chi-square tests for proportions. We hypothesized that a higher proportion of subjects in the AIMS Group would prioritize AIMS behaviors compared to subjects in the Control Group.

**Figure 1:**
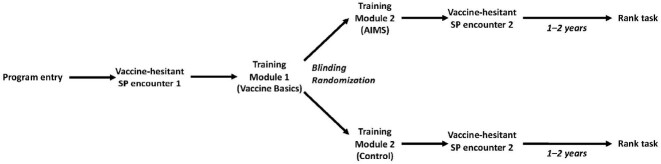
Curriculum Flow Diagram

**Figure 2:**
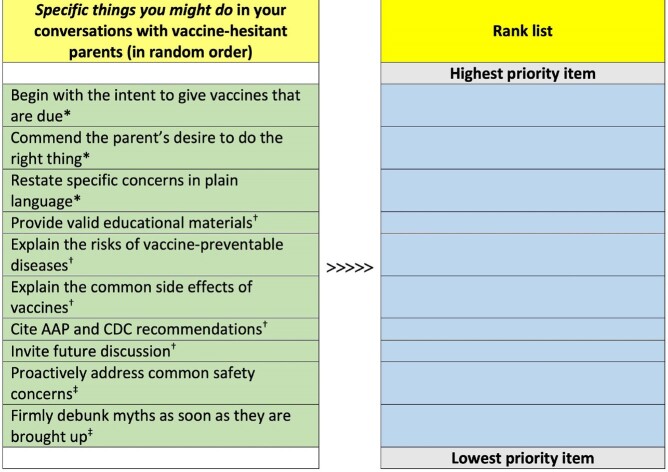
Rank Task

**Results:**

Characteristics of study participants are shown in Figure 3. Seventy-seven percent of the 31 residents in the Validation Group met criteria for prioritization of AIMS behaviors, compared with only 38% of the 32 subjects in the Control Group (*p-*value = 0.003). However, only 19% of the 32 residents in the AIMS Group met criteria for prioritization of AIMS behaviors (*p*-value = 0.164, AIMS Group vs Control Group).

**Figure:**
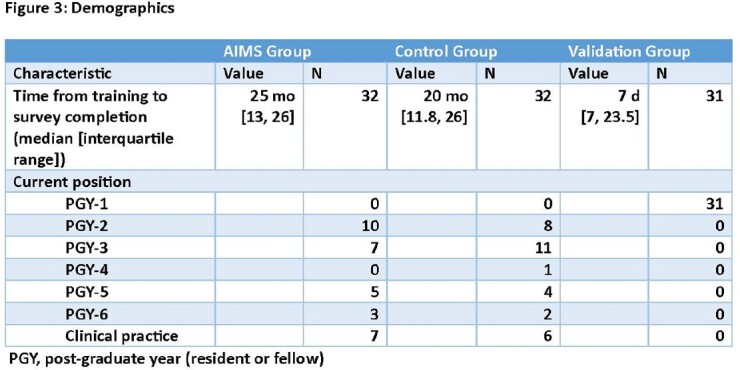

**Conclusion:**

AIMS communication behaviors are ranked highly shortly after training, but this prioritization is not retained over time. Future studies of the utility of structured vaccine hesitancy communication strategies should incorporate repetition and spaced retrieval to enhance retention and ensure deployment in practice.

**Disclosures:**

**Shanna M. Barton, MD, M.Sc.**, Sanofi Pasteur: Grant/Research Support **Gary S. Marshall, MD**, GSK: Advisor/Consultant|GSK: Grant/Research Support|GSK: Honoraria|Merck: Advisor/Consultant|Merck: Grant/Research Support|Merck: Honoraria|Moderna: Advisor/Consultant|Moderna: Grant/Research Support|Moderna: Honoraria|Pfizer: Advisor/Consultant|Pfizer: Grant/Research Support|Pfizer: Honoraria|Sanofi: Advisor/Consultant|Sanofi: Grant/Research Support|Sanofi: Honoraria|Seqirus: Advisor/Consultant|Seqirus: Grant/Research Support|Seqirus: Honoraria

